# Iteration expansion and regional evolution: phylogeography of *Dendrobium officinale* and four related taxa in southern China

**DOI:** 10.1038/srep43525

**Published:** 2017-03-06

**Authors:** Beiwei Hou, Jing Luo, Yusi Zhang, Zhitao Niu, Qingyun Xue, Xiaoyu Ding

**Affiliations:** 1 College of Life Sciences, Nanjing Normal University, Nanjing 210023, China; 2 Nanjing Institute for Comprehensive Utilization of Wild Plants, Nanjing 210042, China; 3 Jiangsu Industrial Technology Research Institute, Nanjing 210042, China

## Abstract

The genus *Dendrobium* was used as a case study to elucidate the evolutionary history of Orchidaceae in the Sino-Japanese Floristic Region (SJFR) and Southeast Asia region. These evolutionary histories remain largely unknown, including the temporal and spatial distribution of the evolutionary events. The present study used nuclear and plastid DNA to determine the phylogeography of *Dendrobium officinale* and four closely related taxa. Plastid DNA haplotype and nuclear data were shown to be discordant, suggesting reticulate evolution drove the species’ diversification. Rapid radiation and genetic drift appeared to drive the evolution of *D. tosaense* and *D. flexicaule*, whereas introgression or hybridization might have been involved in the evolution of *D. scoriarum* and *D. shixingense*. The phylogeographical structure of *D. officinale* revealed that core natural distribution regions might have served as its glacial refuges. In recent years, human disturbances caused its artificial migration and population extinction. The five taxa may have originated from the Nanling Mountains and the Yungui Plateau and then migrated northward or eastward. After the initial iteration expansion, *D. officinale* populations appeared to experience the regional evolutionary patterns in different regions and follow the sequential or rapid decline in gene exchange.

Flowering plants provide superb cases for investigating evolutionary processes, and orchids are particularly useful examples because of their extraordinary diversity^1^. Orchids are derived from one of the largest and most diverse families of flowering plants in the world. Approximately 73 percent of orchids are epiphytic^2^, and epiphytism has more effectively stimulated the development of orchid species richness^3^. However, data on the ecological and genetic processes in epiphytic orchids are limited. The strong genetic drift followed by natural selection has been proposed as the key to the diversification of the Orchidaceae^4^,^5^,^6^. The considerable species diversity in Orchidaceae has been largely explained as a consequence of sequential and rapid interplay between drift and natural selection^6^.

Species richness in plants is correlated with many biological and geohistorical factors, most of which increase ecological opportunities^7^,^8^. In many orchid-rich environments, orchids are typically characterized by small or disjunct populations, and long-distance dispersal lead to low levels of genetic differentiation^9^,^10^. Orchid colonization and genetic drift may potentially increase the speed or likelihood of speciation when coupled with natural selection^11^, and founding events could create a shift in allele frequencies and lead to speciation followed by ongoing genetic drift in certain situations^12^. However, strong evidence showing that genetic drift in isolated populations has played a major role in the diversification of the Orchidaceae is not available.

Historical, ecological, and biogeographic factors are often the major forces that shape global biodiversity because of their influence on regional differences in speciation, migration, and extinction^13^,^14^. The Sino-Japanese Floristic Region (SJFR) includes “Sino-Himalayan Forest” and “Sino-Japanese Forest”, and the associated plant diversity is primarily related to the region’s climatic and physiographical heterogeneity and complex geological history as well as the assumed antiquity of the flora and limited influence of major Quaternary glaciations^11^. However, large-scale phylogeographic comparisons among multiple sympatric plants with similar ecological preferences are still scarce in this region^15^,^16^. In the late Miocene, the monsoon climate began to dominate East Asia^16^. Although orchid species are characterized by dust-like seeds dispersed by wind^17^, the impact of ancient monsoons and orchid expansion in East Asia remains unclear. *Dendrobium* species are the important economic plant and distributed widely in the SJFR and Southeast Asia region, therefore we used the genus *Dendrobium* as a case study to elucidate the evolutionary history of Orchidaceae in these two regions.

As one of the largest genera in Orchidaceae, *Dendrobium* includes 1500–2000 species distributed in the tropical and subtropical regions of Asia and North Australia, where it exhibits distinctive ecological diversification as typical epiphytic orchids^18^. Based on evidence obtained from fossil leaves, *Dendrobium* had colonized New Zealand by the Early Miocene^19^. China has 74 species and two varieties of this genus, of which 40 species have been used in traditional medicines for many centuries, and all these species are distributed in the SJFR^20^,^21^. In particular, the stem of *Dendrobium officinale* Kimura et Migo is an ingredient of “Tiepi Fengdou”^22^,^23^, which is ranked “the first of the nine kinds of supernatural Chinese medicinal herbs.”

*D. officinale* is an insect-pollinated, predominantly outcrossing species. Historically, *D. officinale* has been distributed widely throughout the moderately damp mountain habitats of the subtropical and warm temperate zones in China, which is naturally fragmented, at altitudes of 800–1600 m^20^,^24^. Unfortunately, the current distribution is highly fragmented and discontinuous, and many *D. officinale* populations previously recorded have been extirpated^25^,^26^. Based on the molecular systematics of *Dendrobium, D. officinale* is sister to *D. tosaense* Makino; *D. flexicaule* Z. H. Tsi, S. C. Sun & L. G. Xu; and *D. scoriarum* W. W. Smith (or *D. scoriarum* S. J. Cheng & C. Z. Tang). Accordingly, these four species were proposed for inclusion in the Catenatum group^27^, which has been well supported by both molecular and morphological evidence. The novel species *Dendrobium shixingense* sp. nov. was recently reported^28^, and it is considered most similar to *D. scoriarum, D. flexicaule* and *D. officinale*. Based on previous research^23^,^27^, these five species are closely related taxa; however, their evolutionary relationship remains to be clarified.

The recovered intraspecific divergences and phylogeographic patterns of multiple-species, especially the closely related species or a monophyletic group, may allow us to better understand the high plant diversity^29^. In this study, we sought to elucidate the evolutionary and population demographic history of *D. officinale, D. tosaense, D. flexicaule, D. scoriarum* and *D. shixingense*. To determine the evolutionary history and expansion patterns of Orchidaceae in the SJFR and Southeast Asia region, the study had three specific objectives: 1) to illustrate the expansion model of *D. officinale* and infer the influence of the glacial period, 2) to determine the phylogeographical structure of *D. officinale* and explain the cause of its endangered status, and 3) to clarify the relationship between *Dendrobium* and its four relatives and elucidate the evolutionary history of five *Dendrobium* species.

## Results

### CpDNA variation

**Figure 1:**
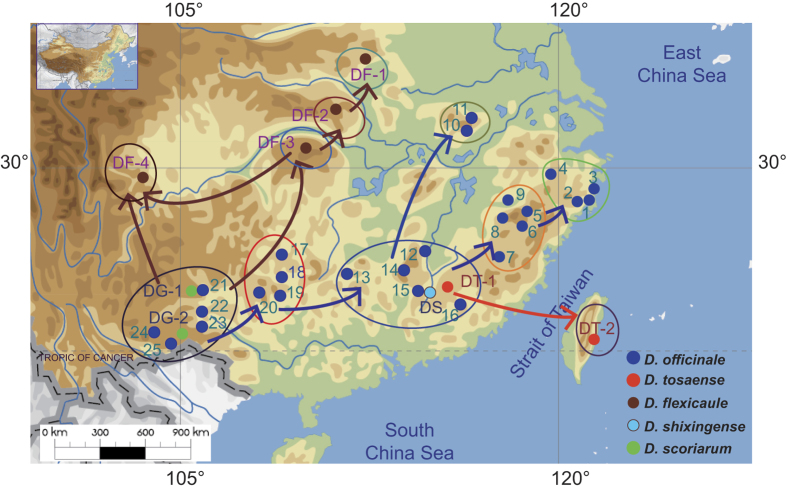
Map of sampling localities and migration routes for 34 populations of *Dendrobium officinale* and four related taxa in 11 different regions in China and the island of Taiwan. pop 1–4, Yandang Mts.; pop 5–9, Wuyishan Mts.; pop 10–11, Dabieshan Mts.; pop 12–16, DT-1 and DS, Nanling Mts.; pop 17–20, East Yungui Plateau; pop 21–25, DG 1–2, Yungui Plateau; DT-2, Taiwan; DF-1, Funiushan Mts.; DF-2, Daba Mts.; DF-3, Wuling Mts.; DF-4, East Hengduan Mts. The map was drawn using ArcGIS 10.2 (ESRI, CA, USA) and Adobe Illustrator CS3 13.0.

We sequenced three cpDNA-intergenic spacer (IGS) regions with a total length of 2225 bp for 435 accessions from 32 populations representing *D. Officinale* and the four closely related taxa (Fig. 1, Tables S1). The results indicated that nucleotide substitutions occurred at 52 sites and one inversion (7 bp) was present in the *acc*D-*psa*I region. Indel mutations occurred in only two species, with one (9 bp) occurring in *D. flexicaule* and two occurring in *D. scoriarum* (Tables S2). A total of 34 haplotypes were identified in five *Dendrobium* species, with 25 from *D. officinale*, 2 from *D. shixingense*, 3 from *D. scoriarum*, 3 from *D. flexicaule*, and 2 from *D. tosaense* (one haplotype was shared by *D. tosaense* and *D. officinale*).

**Figure 2:**
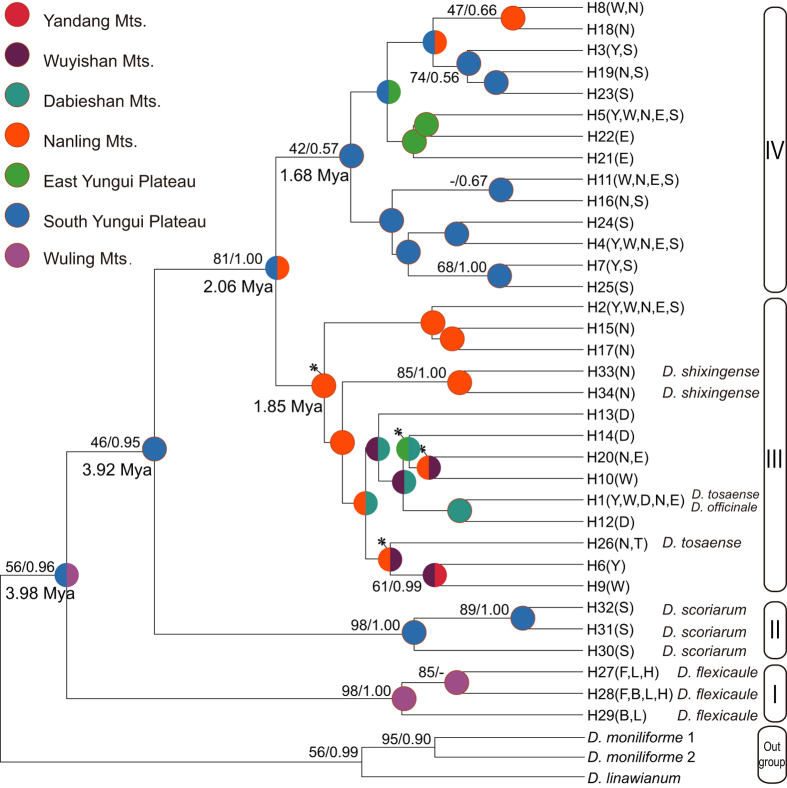
Bayesian inference tree of 34 chloroplast DNA haplotypes from *Dendrobium officinale* and four related taxa, and statistical reconstructions (Pie charts) of ancestral areas based on the Bayes-DIVA analyses of cpDNA. Support values (ML bootstrap/Bayesian posterior probability) and divergence time were shown at nodes. Haplotypes of different species are indicated in the tree terminals. Eleven major distributional regions for *D. officinale* and four related taxa were Yandang Mts. (Y), Wuyishan Mts. (W), Dabieshan Mts. (D), Nanling Mts. (N), East Yungui Plateau (E), Yungui Plateau (G), Taiwan (T), Funiushan Mts. (F), Daba Mts. (B), Wuling Mts. (L) and East Hengduan Mts. (H). Ancestral areas with probability <0.05 are marked with asterisks.

Based on Modeltest 3.7 test, the GTR + I + G model was chosen as the best fit for each data partition for the two phylogenetic analyses. The phylogenetic trees inferred from Maximum likelihood (ML) and Bayesian inference (BI) analyses were nearly identical with high support values and included two outgroup taxa (Fig. 2). All 34 cpDNA haplotypes were clustered into four clades: Clade I contained three haplotypes from *D. flexicaule*, Clade II contained three haplotypes from *D. scoriarum*, and Clades III and IV contained the other 28 haplotypes, with haplotypes from *D. shixingense* and *D. tosaense* included in Clade III and haplotypes from *D. officinale* falling into Clade IV.

**Figure 3:**
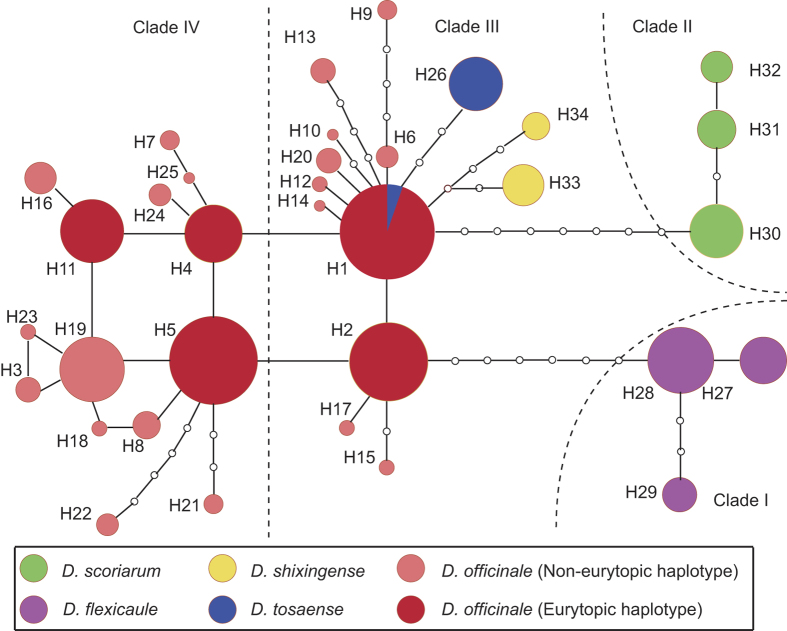
Network analysis of genealogical relationships among the 34 chloroplast DNA haplotypes of *Dendrobium officinale* and four related taxa.

**Figure 4:**
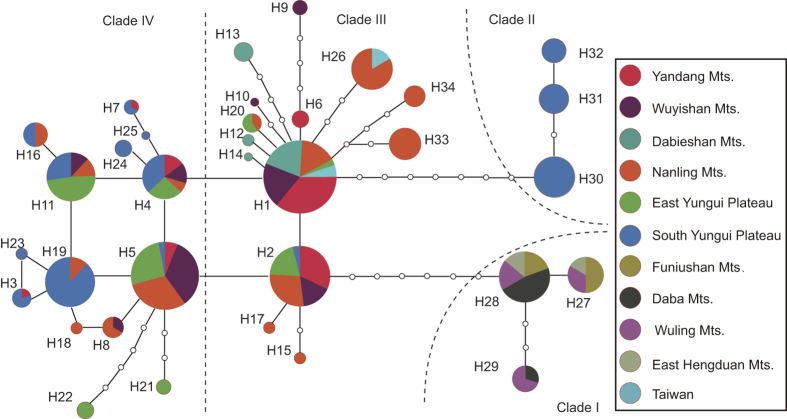
Summary of cpDNA haplotypes in 11 distributional regions of *D. officinale* and four relative taxa.

A median-joining analysis revealed a multi-species haplotype network that was congruent with the gene tree, with all four clades recovered (Fig. 3). In *D. officinale*, haplotype H1 in Clade III was an ancestral haplotype, and a one-step mutation produced haplotype H2. Haplotype H1 interrelated and interacted with three related taxa, and haplotype H2 was separated from haplotype H28 of *D. flexicaule* by seven mutational steps. These two haplotypes had high frequencies in Clade III, whereas Clade IV had four high frequency haplotypes: H4, H5, H11 and H19. In the two clades of *D. officinale*, all high frequency haplotypes except H19 were eurytopic haplotypes that occurred in almost all investigated regions (Fig. 4, Tables S3), whereas H19 was present only in the Nanling Mountains and the South Yungui Plateau. Haplotypes of Clade III mainly occurred in the populations of the three eastern regions, and the haplotypes of Clade IV mainly occurred in the populations of the three western regions. Compared with the other haplotypes, H3 and H7 had a distinct geographical distribution, with the former occurring only in the Yandang Mountains located in northeastern China and the latter occurring only in the South Yungui Plateau in southwestern China.

### Internal transcribed spacer (ITS) variation

**Figure 5:**
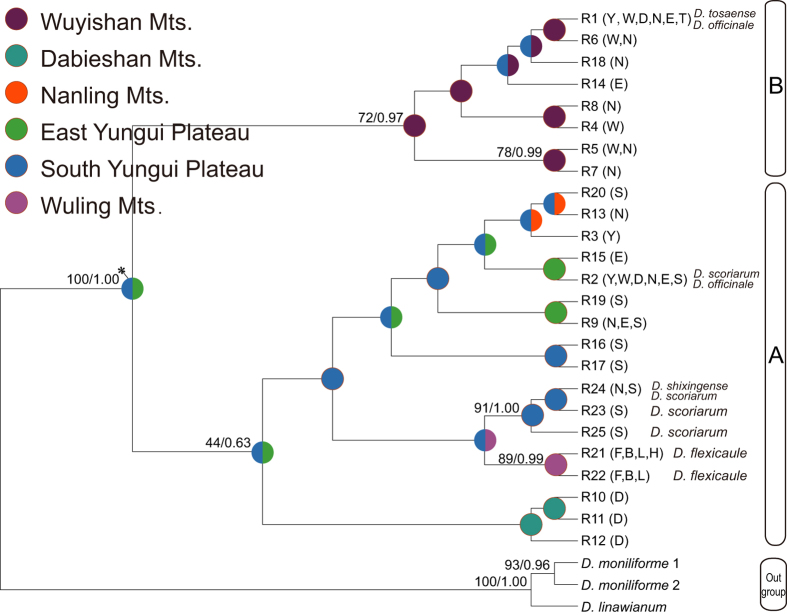
Bayesian inference tree of 25 ITS ribotypes from *Dendrobium officinale* and four related taxa, and statistical reconstructions (Pie charts) of ancestral areas based on the Bayes-DIVA analyses of ITS data. Support values (ML bootstrap/Bayesian posterior probability) and divergence time were shown at nodes. ITS ribotypes of different species are indicated in the tree terminals. Eleven major distributional regions for *D. officinale* and four related taxa were Yandang Mts. (Y), Wuyishan Mts. (W), Dabieshan Mts. (D), Nanling Mts. (N), East Yungui Plateau (E), Yungui Plateau (G), Taiwan (T), Funiushan Mts. (F), Daba Mts. (B), Wuling Mts. (L) and East Hengduan Mts. (H). Ancestral areas with probability <0.05 are marked with asterisks.

**Figure 6:**
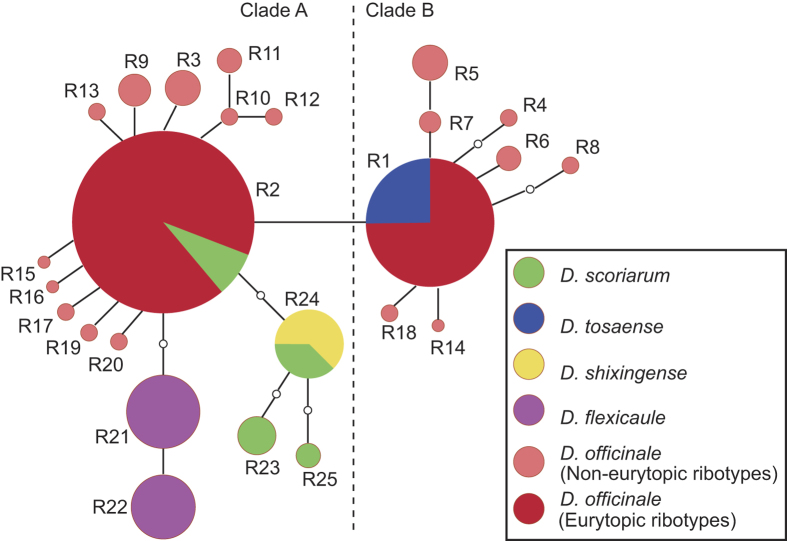
Network analysis of genealogical relationships among the 25 ITS ribotypes of *Dendrobium officinale* and four related taxa.

For the ITS regions, 25 ribotypes were identified in *D. officinale* and its closely related species, including 20 from *D. officinale*, 1 (R24) from *D. shixingense*, 4 (R2, R23, R24 and R25) from *D. scoriarum*, 2 (R21 and R22) from *D. flexicaule*, and 1 (R1) from *D. tosaense* (ribotype R1 of *D. tosaense* and ribotype R2 of *D. scoriarum* (R2) were both shared with *D. officinale*, and ribotype R24 of *D. shixingense* was shared with *D. scoriarum* (Figs 5 and 6)). Within a 578-bp-long ITS region, a total of 29 base substitutions were detected in 25 ribotypes.

**Figure 7:**
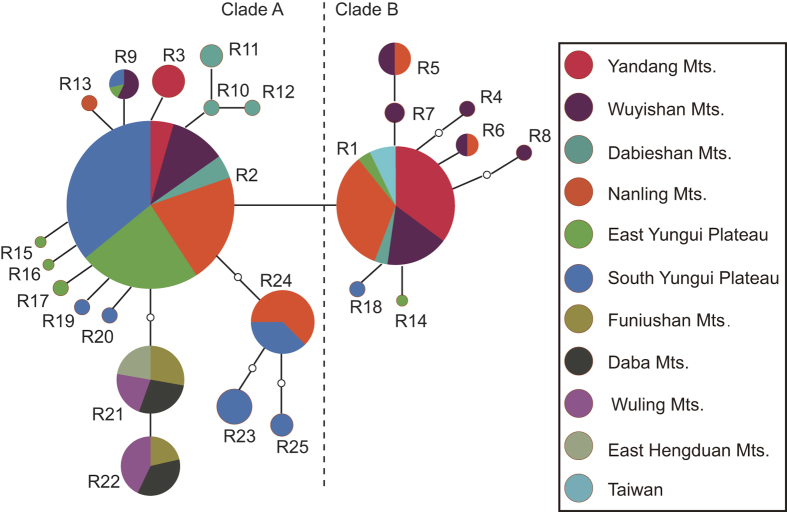
Summary of ITS ribotypes in 11 distributional regions of D. officinale and four relative taxa.

Low genetic variation and shared ribotypes were detected in the rDNA. The two phylogenetic analyses generated topologically similar trees (Fig. 5), with all 25 ribotypes clustering into two clades (A and B). The ITS ribotypes of three related species were all nested in various positions of *D. officinale*. Two ribotypes (R21 and R22) of *D. flexicaule* were sisters to three ribotypes of *D. scoriarum* in Clade A. For *D. officinale*, R1 and R2 were high-frequency ribotypes showing continuity in their geographical distributions (Fig. 7, Tables S4). Haplotypes of Clade A mainly occurred in the populations of the two western regions and the Dabieshan Mountains, and the haplotypes of Clade B mainly occurred in the populations of the Wuyishan Mountains.

### Phylogenetic divergence time estimation

Compared with the three outgroups, significant heterogeneity was not detected among the cpDNA haplotypes of the five species by relative rate tests (all *P* > 0.05). The corrected cpDNA sequence divergence (*d*A) between *D. flexicaule* and the other taxa was 0.00594, the corresponding estimate between *D. scoriarum* and *D. officinale* was 0.00600, and the intraspecific divergence of *D. officinale* was markedly lower at 0.00298. The substitution rate of the combined sequences (μ) was consistently estimated at 1.54 × 10^−9^ substitutions per site per year (s/s/y) based on the length used for the pairwise analyses. Based on the substitution rate, the intraspecific divergence of *D. officinale* was dated to *c*. 2.06 Mya. However, the interspecific divergences of *D. flexicaule* and *D. scoriarum* were dated to *c*. 3.98 Mya and *c*. 3.92 Mya, respectively (Fig. 2), indicating that they were compatible with the Pliocene event.

### Biogeographic analyses

The Bayesian dispersal-vicariance analysis (Bayes-DIVA analyses) of the cpDNA data suggested that the South Yungui Plateau was the ancestral region for *D. officinale* and its related taxa (Figs 1 and 2). The South Yungui Plateau is the most likely origin center for these species, and multiple dispersals might have occurred from the Yungui Plateau to the Nanling Mountains and then to the Dabieshan Mountains, the Wuyishan Mountains, the Yandang Mountains, and the Taiwan region. For *D. flexicaule*, a second northward distribution channel occurred from the South Yungui Plateau to the Wuling Mountains and the East Hengduan Mountains and then to the Daba Mountains and the Funiushan Mountains. Consistently, the Bayes-DIVA analyses of the nrITS data resulted in similar reconstructions (Fig. 5), although they did not show a distinct vicariance signal.

### Intraspecific differentiation

Twenty-five haplotypes were identified by comparing the three intergenic spacer regions of the cpDNA of 343 individuals in 25 populations of *D. officinale*. The cpDNA data revealed high estimates of haplotype diversity (*h*_T_ = 0.875) and nucleotide diversity (*π*_T_ = 0.00093) but did not indicate a clear geographical pattern of within-population diversity. A significantly larger N_ST_ than G_ST_ value was estimated across all populations for the cpDNA datasets, which indicated that the genetic variation of *D. officinale* was geographically structured across its distributional regions (Table 1). Six different mountain regions were similar in the level of genetic diversity and the number of chlorotypes or ribotypes, whereas the Dabieshan Mountains had the lowest number of chlorotypes and genetic diversity (Fig. 4, Tables S3). Non-hierarchical AMOVAs for *D. officinale* revealed low levels of divergence among regions in both cpDNA and ITS sequences (Table 2). For the cpDNA data, 34.02% of the total genetic variation was partitioned by population and 16.94% was partitioned by region, whereas for the ITS data, only 29.76% of the total variation was partitioned among the populations.

### Recent demographic events

The topological structure of 25 *D. officinale* haplotypes was the same as that for all haplotypes with two branches. Demographic analyses (mismatch distribution analyses (MDAs)) were conducted for two cpDNA-IGS lineages of *D. officinale*. The mismatch distributions were unimodal for *D. officinale* and its Clades III and IV (Appendix S3), indicating that the population underwent a recent expansion. Spatial and demographic expansions were suggested by uniformly insignificant SSD and HRag values; however, expansions were not supported by insignificant Tajima’s D and Fu’s FS values (Table 3).

## Discussion

Species evolution can be initiated by phenotypic differences upon local environmental selection, which can ultimately lead to genetic differentiation^30^,^31^. Previous studies on orchids confirmed that certain species showed consistent distinctions at morphological, ecological and geographical levels without presenting genetic differentiation^31^. In the current research, *D. tosaense* and *D. officinale* shared chloroplast haplotypes as well as ribotypes, and genetic differences were not observed. Nonetheless, as revealed in a previous edition of Flora of China^20^, the above two species have phenotypic differences in the color of their sepals and petals and morphological and structural differencesin the lip. Orchids are typically characterized by low levels of population genetic differentiation. Phenotypic variations may not always reflect real genetic variations^32^. For *D. tosaense*, exiguous phenotypic differences between two species could have resulted from rapid radiation^33^. The island of Taiwan did not separate from the Asian continent until the mid-Pleistocene, and the vegetation distribution in East Asia was continuous during the Last Glacial Maximum (LGM)^34^. Consistent with findings in previous studies^35^,^36^, *D. tosaense* might have expanded southeastward from the Asian continent to the island of Taiwan during a sea-level depression but failed to disperse to Japan and South Korea because of the climatic conditions of these regions.

Gene flow barriers are considered the most definitive evidence for species delimitations^32^. Compared with *D. officinale, D. flexicaule* resides at higher latitudes and is not in sympatric distribution with the other related species, which might have reduced the levels of interspecific gene flow. The ITS ribotypes of *D. flexicaule* were shown to be nested within those of *D. officinale*, which might have been caused by genetic drift. Although haplotypes of *D. flexicaule* are derived from H2, the eurytopic haplotype H2 of *D. officinale* was not found in the Dabieshan Mountains, suggesting that the *D. flexicaule* populations sampled in the Funiushan Mountains and the Daba Mountains in this study did not originate from the Dabieshan Mountains. Based on the Bayes-DIVA analyses, evolution of *D. flexicaule* appeared to involve the *D. officinale* populations distributed in the South Yungui Plateau and the East Yungui Plateau, with a northward distribution channel extending from the Yungui Plateau to the Wuling Mountains and the East Hengduan Mountains and then to the Daba Mountains and the Funiushan Mountains. The evolution of interspecific reproductive barriers was existed between *D. flexicaule* at higher latitudes and *D. officinale* at lower latitudes. Under high average genetic differentiation, genetic drift might have been the driving forces underlying the evolution of *D. flexicaule*.

Secondary hybridization and introgression have been observed in marsh orchid (*Dactylorhiza* Necker *ex* Nevski)^37^. In this study, *D. officinale, D. scoriarum* and *D. shixingense* were all sympatric species. However, their phylogenetic relationships inferred from cpDNA sequences were inconsistent with those derived from nuclear ITS sequences, three species had a diverse mixture lineages. For example, *D. scoriarum* and *D. officinale* shared the ancestral (interior) ribotypes but not the same haplotypes. The sharing of the ancestral ribotypes R1 might be interpreted as persistent sharing of ancestral polymorphism due to the young age of lineages or as a consequence of recurrent gene flow between two speices. Moreover, species sharing of haplotypes could be caused by incomplete lineage sorting preferentially involves ancestral haplotypes, while sharing of recently derived (tip) haplotypes was more likely to arise through introgression^38^. In addition, genetic differentiation of Clade III was dated to *c*.1.85 Mya, the interspecific divergences of *D. scoriarum* and *D. shixingense* had a long time lag with 2.07 Mya. The haplotypes of *D. shixingense* originated from *D. officinale* with only three or four step mutations, whereas its ancestral ribotypes were shared with *D. scoriarum*. Such discordances might be attributed to gene introgression which drove the evolution of *D. shixingense*, and hybridization might also play a major role in its evolution.

A number of plant groups have shown ‘cytonuclear discordance’ and reticulation in their phylogenetic networks, which is evidence of reticulate evolution^39^,^40^. Reticulate evolution has been shown to be a driving force for species diversification via hybridization events^41^. In addition, transitions between self-compatibility (SC) and self-incompatibility (SI) can gradual accumulate genetic incompatibilities preventing gene flow among *Dendrobium* species^42^. For *D. officinale* and its related taxa, the discordance between gene trees and the network analysis indicated that reticulate evolution occurred in these five species. Gene flow between species via introgression is a common event, with the genomes of many species apparently permeable to alleles from related species^43^. Alternatively, gene flow between species may have hindered speciation^44^,^45^. The occurrence of gene flow in these five species suggested that hybridization or introgression can promote efforts to the evolutionary history of *Dendrobium*. Differentiation among these five species might have been caused by drift and selection pressures, although prolonged and strong drift likely did not occur. During geological events and climatic shifts, closely related taxa adapted to different florescence and habitats^20^,^28^. Such as *D. officinale, D. tosaense, D. scoriarum, D. flexicaule* an*d D. shixingense* are lithophytic on rocks or tree trunks in mountain valleys at different altitudes, ca. 800–1600 m, 300–1200 m, 1200–1500 m, 1200–2000 m and 400–600 m separately. The occurrence of positive selection highlights the importance of adaptive factors in *Dendrobium* evolution.

In this study, the haplotype network of *D. officinale* displayed a stellate framework, thus indicating the occurrence of expansion events in *D. officinale*^46^. However, insignificant Tajima’s D values yielded from the mismatch analysis did not support a recent population expansion; hence, the expansion events in *D. officinale* might have occurred at the early stage of its evolutionary history. High-frequency haplotypes and ribotypes were present in almost all sampled regions, which presented similar haplotype diversity. Eurytopic haplotypes observed in both branches of *D. officinale* indicated primary or iteration expansion events that occurred at an early stage of evolutionary history. The divergence of the two branches of *D. officinale* was dated to *c*. 2.06 Mya, and the expansion of haplotypes in Clades IV preceded that of Clades IV. Under iteration expansion, 5 haplotypes of *D. officinale* represent the eurytopic haplotypes that expanded to almost all investigated regions.

In the six distributional regions of *D. officinale*, low haplotype diversity occurred in eastern (the Yandang Mountains and the Wuyishan Mountains) and northern China (the Dabie Mountains), whereas high haplotype diversity occurred in the Nanling Mountains and the South Yungui Plateau, implying that these regions and their adjacent areas could be recognized as the diversity centers of *D. officinale*. Based on the results of the Bayes-DIVA analyses, the ancestral region of *D. officinale* may be the South Yungui Plateau, which is adjacent to the Qinghai-Tibetan Plateau (QTP), one of the world’s biodiversity hotspots^47^. *D. scoriarum* and *D. officinale* experienced initial genetic divergence in the South Yungui Plateau, whereas *D. shixingense* underwent hybridization or introgression events in the Nanling Mountains. These lines of evidence suggest that the Nanling Mountains and the South Yungui Plateau may be the origin centers of the four closely related taxa. The Nanling Mountains, which stretch west to east in southern China, is also known as the Nanling Corridor and represents one of the most important corridors for the eastward migration of East Asiatic flora^48^,^49^. Through the Nanling Corridor, *D. officinale* underwent range migration toward the east to the Yandang Mountains and the Wuyishan Mountains, whereas *D. tosaense* migrated toward the Taiwan region. However, *D. officinale* also expanded northward from the Nanling Mountains to the Dabie Mountains. Overall, the dispersal of *D. officinale* showed a trend towards the east and the north, which is consistent with previous genetic structure studies of *D. officinale*^50^,^51^.

According to previous studies, multiple glacial (cryptic) refuges occurred in southern China^52^,^53^,^54^,^55^,^56^, and the “refugia-within-refugia” scenario was described for the refuges *of Eurycorymbus cavaleriei* in subtropical China^49^. *D. officinale* and its related taxa experienced interspecific divergence before the glacial period in the Pleistocene^57^, and the intraspecific divergence of *D. officinale* occurred at *c*. 2.06 Mya. For 20 non-eurytopic haplotypes of *D. officinale*, 14 haplotypes were private haplotypes for six regions, and each region contained 1–3 private haplotypes. The phylogeographical structure of *D. officinale* is consistent with paleovegetation reconstructions, suggesting that *D. officinale* did not experience dramatic range fragmentation into separate glacial refugia during the LGM. Based on fossil-pollen data, southern China was not covered in a uniform ice sheet but rather a temperate deciduous forest during the LGM^15^,^34^,^58^. Thus, core natural distribution regions might have represented glacial refuges for *D. officinale* and its related taxa.

In the two haplotype clades, five eurytopic haplotypes occurred in almost all sampled regions, whereas the other haplotypes revealed distinct regional characteristics, thus indicating that eurytopic haplotypes underwent iteration expansion events at an early stage of the evolution history and then experienced a decline in gene exchange. In the long stage of its evolutionary history, *D. officinale* have adapt to both high- and low-latitude regions, and the habitats in the low-latitude regions varies according to geographical features. For example, the Yungui Plateau presented limestone and montane forest trunk habitats and the Nanling Mountains presented Danxia landform habitats suitable for the growth of epiphytic *D. officinale*, and the Yandang Mountains and the Wuyishan Mountains in eastern China presented acidic volcanic or granite surfaces. For Orchidaceae, the main challenges to survival were related to complex interactions among mycorrhizal fungi, pollinators and host trees^59^. Similarly, the divergence of two *Dendrobium* species (*D. speciosum* and *D. tetragonum*) were also driven by topographical and climatic conditions in eastern Australia^60^. Therefore, after the initial iteration expansion, *D. officinale* evolved independently in different regions and subsequently experienced gene flow events. Although each of the related taxa experienced restricted distribution because of habitat selection pressure, the four taxa related to *D. officinale* differed in their regional distributions and evolution.

Disrupted gene flow is known to have a greater impact on fragmented populations than on continuous populations. Artificial cultivation experiments showed that snails, slugs and mice are the natural enemies of *Dendrobium* species. As lithophytic, *D. officinale* disjunctly distributed at high altitude mountain range. In this research, 25 sampled populations of *D. officinale* were distributed in six regions that reflected fragmented habitation. Although orchids are typically characterized by small, disjunct populations^6^,^61^,^62^, the mountain habitat fragmentation of *D. officinale* is currently severe, and many extant populations have been deteriorating. A large number of haplotypes restricted to different regions indicate that the populations were naturally isolated for long periods. The divergence age between haplotypesis was consistent with historical divergence events and not recent fragmentation. Although anthropogenically driven habitat fragmentation was pervasive in the region^63^,^64^,^65^, human over-exploitation likely did not cause the habitat fragmentation associated with *D. officinale*; however, human activity could have led to extinction events.

Similar to most orchids, many thousands of dust-like *D. officinale* seeds can be dispersed over long distances by typhoons, tourbillions or tornadoes^50^,^66^. Among the haplotypes in Clade IV, only haplotypes H3 and H7 were found in both the South Yungui Plateau and the Yandang Mountains, two relatively distant regions. Haplotype H7 and H3 evolved from H4 and H5 by three mutational steps separately. Haplotype H4 and H5 occurred in almost in all sampled regions containing Yandang Mountains. Haplotype H3 and H7 could be the traces of historical evolution of Clade IV in Yandang Mountains. On the other hand, the seeds harboring these haplotype likely could not disperse by monsoon from the southwestern population to the eastern region of the Yandang Mountains. Since the early twentieth century, peasants in the Yandang Mountain region have been collecting and processing *D. officinale* across China, which also afforded an opportunity for its artificial migration. Hence, human activities may have played a key role in shaping the distributional patterns of *D. officinale* over the past 100 years, but the level of artificial migration^67^ should be evaluated migration rate by genetic data analysis in the future.

In conculsion, *Dendrobium* species have been used as traditional medicines for many centuries in China; however, many of them have become endangered. In this study, phylogeographic and systematic analyses were performed to clarify the evolutionary history of *D. officinale* and its closely related taxa and identify the reasons for their endangered status in southern China. Rapid radiation and genetic drift appear to be the driving force of the evolution of *D. flexicaule* and *D. tosaense*, whereas hybridization and introgression might have been involved in the evolution of *D. scoriarum* and *D. shixingense*. The phylogeographical structure suggests that *D. officinale* did not experience dramatic range fragmentation into separate glacial refugia during the LGM. After the early stage of its iteration expansion, *D. officinale* experienced independent evolutionary events in different regions and then a decline in gene exchange. The four closely related taxa originated in the South Yungui Plateau and the Nanling Mountains and then migrated northward to the Wuling Mountains, the East Hengduan Mountains, the Daba Mountains, and the Funiushan Mountains. From the Nanling Mountains, the species expanded northward to the Dabie Mountains, eastward to the Wuyishan Mountains and the Yandang Mountains, and then to the Taiwan region. Although *D. officinale* has experienced natural habitat fragmentation for long periods, human disturbance was responsible for its migration and population extinction in recent years. Hence, the genetic drift and introgression accounted for the evolutionary history of *D. officinale* and four related *Dendrobium* species, whereas reticulate evolution drove the species’ diversification.

## Materials and Methods

### Sampling and DNA extraction

Between 2009 and 2013, a total of 499 individual plants were sampled from 34 populations belonging to five *Dendrobium* species distributed in 11 different regions of China (Fig. 1, Tables S1). The samples were stored in silica gel. Because of the endangered status of *D. officinale*, sampling at each of these localities was limited to 20 individuals. Among the five species, four populations of *D. flexicaule* (DF-NZ, DF-SN, DF-ES and DF-GL) and one population of *D. tosaense* (DT-TW) inallopatry with *D. officinale* were sourced from the Funiushan Mountains, the Daba Mountains, the Wuling Mountains, the East Hengduan Mountains and the Taiwan region, respectively. *D. officinale* samples were collected from six regions: the Yandang Mountains, the Wuyishan Mountains, the Dabieshan Mountains, the Nanling Mountains, the East Yungui Plateau, and the South Yungui Plateau. In sympatry with *D. officinale, D. scoriarum* populations XY-DG and WS-DG were from the South Yungui Plateau, and both the *D. tosaense* population LN-DT and the *D. shixingense* population SX-DS were from the Nanling Mountains.

Voucher specimens of representatives of all sampled taxa and populations were stored at the Herbarium of Nanjing Normal University. For the phylogenetic DNA analysis, three accessions from two species of *Dendrobium* were arbitrarily designated as outgroups: *Dendrobium linawianum* Rohb. f. and *Dendrobium moniliforme* (Linn.) Sw., which were collected from the Guangxi Zhuang Autonomous Region and the Yunnan Province of China. Genomic DNA was extracted from each sample using a standard CTAB DNA extraction protocol modified by adding 2% PVP-40 to the buffer^68^.

### Nuclear and chloroplastDNA sequencing

Nuclear ITS regions and three noncoding chloroplast fragments (*acc*D-*psa*I, *trn*C-*pet*N, and *rps*15-*ycf*1) were amplified from 475 samples of *D. officinale* and the related taxa, with an average of 14.8 samples per population (Tables S1 and S5). Polymerase chain reactions (PCRs) were conducted in 25 μL reaction mixtures that each contained 20–50 ng of genomic DNA as a template, 1× PCR buffer (TaKaRa, Japan), 2 mM MgCl_2_, 200 μM dNTP (TaKaRa, Japan), 0.2 μM of each primer, and 0.5 units Taq DNA polymerase (TaKaRa, Japan). PCR amplification was performed with a Peltier Thermal Cycler PTC-200 (BIO-RAD) according to the following program: initial denaturation at 94 °C for 5 min, followed by 30 cycles of denaturation at 94 °C for 1 min, annealing at 55 °C for 1 min, and extension at 72 °C for 2 min, and then a final extension at 72 °C for 10 min. The purified PCR products from single bands were directly sequenced by ABI Prism BigDye Terminator version 3.1 Cycle Sequencing Kit with the PCR primers described above and then by the ABI 3130 XL sequencer (Applied Biosystems).

All sequences were aligned using MUSCLE in MEGA5 except for ambiguous loci^69^. During the analyses, all mononucleotide repeats were excluded because of their highly variable evolutionary rates in the microsatellite regions. Indels and inversions were treated as single mutation events. Haplotypes and ITS ribotypes were identified and distinguished using DnaSP version 4.0^70^. A new haplotype or ITS ribotype was considered authentic when identified in at least three independent sequences. Unique haplotype and ITS ribotype sequences were submitted to GenBank under the assigned accession numbers (Table S6).

### Phylogeographic analyses

Phylogenetic analyses of the cpDNA haplotypes and ITS ribotypes of *D. officinale* and its related taxa were assessed via maximum likelihood (ML) and Bayesian inference (BI) analyses. Modeltest 3.7 was used to select the best nucleotide substitution model under the Akaike information criterion (AIC)^71^. An ML tree was obtained using raxmlGUI version 1.3, which is available at http://sourceforge.net/projects/raxmlgui/. Nodal support values were estimated from 1000 nonparametric bootstrap replicates. MrBayes version 3.1.2 was used to implement the BI analyses^72^. After the program was set with four Markov chains for each analysis and default heating values, the program ran for 10 million generations, with trees sampled every 1000 generations. The first 5000 sampled trees were discarded as burn-in. In addition, we used NETWORK version 4.5 to build a median-joining network (MJN) to visualize the phylogenetic relationships among the cpDNA haplotypes and ITS ribotypes^73^.

Bayes-DIVA analyses were conducted using RASP v.2.0b (Reconstruct Ancestral State in Phylogenies, http://mnh.scu.edu.cn/soft/blog/RASP) to infer the biogeographic history of *D. officinale* and related taxa based on the phylogeny constructed from the cpDNA and nrITS^74^. In this analysis, the sampled populations were distributed in 11 regions: (Y) Yandang Mountains (*D. officinale* populations 01–04); (W) Wuyishan Mountains (*D. officinale* populations 05–09); (D) Dabieshan Mountains (*D. officinale* populations 10–11); (N) Nanling Mountains (*D. officinale* populations 12–16, *D. tosaense* population DT-1 and *D. shixingense* DS); (E) East Yungui Plateau (*D. officinale* populations 17–20); (S) South Yungui Plateau (*D. officinale* populations 21–25 and *D. scoriarum* populations DG-1 and DG-2); Taiwan (T) (*D. tosaense* population DT-2); (F) Funiushan Mountains (*D. flexicaule* population DF-1); (B) Daba Mountains (*D. flexicaule* population DF-2); (L) Wuling Mountains (*D. flexicaule* population DF-3); and (H) East Hengduan Mountains (*D. flexicaule* population DF-4) (Fig. 2). We loaded 10,001 trees previously generated by MrBayes version 3.1.2 and chose the F81 model for the Bayesian MCMC analyses^72^ to allow for different rates of change among the ancestral areas.

### Population genetic analyses

DnaSP version 4.0 was used to calculate the DNA haplotypes (*h*) and nucleotide (π) diversities^70^. Population gene diversities (HS, HT) and between-population divergences (GST, NST) were estimated within each region using the program PERMUT with 1000 permutation tests, and the significant differences observed between the NST and GST may suggest a significant phylogeographic structure^75^. For each dataset (cpDNA and ITS), population differentiation was also quantified with a non-hierarchical analysis of molecular variance by regions using Arlequin version 3.5^76^.

For the cpDNA lineages of *D. officinale*, a mismatch distribution analysis (MDA)^77^ was conducted to test the demographic expansion using Arlequin. The goodness-of-fit based on the sum of squared deviations (SSD) and raggedness index (HRag) was tested using 1000 parametric bootstraps^76^ for each model. In addition, Tajima’s D^78^ and Fu’s F statistics^79^ were employed to test for selective neutrality to infer potential population growth and expansion using Arlequin version 3.5^76^. The statistical significance was tested with 10, 000 permutations.

The rateconstancy of the cpDNA haplotype evolution in *D. officinale* and related taxa was evaluated by the relative rate tests in MEGA^78^ using *D. linawianum* and *D. moniliforme* as outgroups. After determining the rateconstancy, the same program was utilized to infer time since divergence (*T*) of the haplotype lineages from their net pairwise sequence divergence per base pair (*d*_A_) under the Kimura two-parameter model, which corrected for within-lineage diversity. Divergence time was calculated according to the formula *T* *=* *d*_A_/2 μ, where μ is the rate of nucleotide substitution^80^. The mutation rate (μ) was estimated using BEAST 1.7.5^81^. XML files for the BEAST analyses were generated in BEAUti version 1.7.5 with the following settings: GTR + I + G as the substitution model and a piecewise-constant Bayesian skyline tree prior model. Markov chains were run for 10, 000, 000 generations, and every 1000 generations were sampled with at least a 10% burn-in phase.

## Additional Information

**How to cite this article**: Hou, B. *et al*. Iteration expansion and regional evolution: phylogeography of Dendrobium officinale and four related taxa in southern China. *Sci. Rep.* **7**, 43525; doi: 10.1038/srep43525 (2017).

**Publisher's note:** Springer Nature remains neutral with regard to jurisdictional claims in published maps and institutional affiliations.

## Supplementary Material

Supplementary Information

## Figures and Tables

**Table 1:** Estimates of average gene diversity within populations (H_S_) of *D. officinale*, total gene diversity (H_T_), interpopulation differentiation (G_ST_), and the number of substitution types (N_ST_) for chlorotypes and ribotypes across regions.

Region	Pop No	Plant No	cpDNA					ITS				
			n	H_S_	H_T_	G_ST_	N_ST_	n	H_S_	H_T_	G_ST_	N_ST_
Yandang Mts.	4	57	7	0.667	0.696	0.042	0.059	3	0.473	0.490	0.036	0.081
Wuyishan Mts.	5	60	8	0.642	0.824	0.220	0.275	8	0.290	0.613	0.526	0.355
Dabieshan Mts.	2	22	4	0.488	0.600	0.186	0.147	5	0.507	1.000	0.493	0.281
Nanling Mts.	5	72	12	0.676	0.880	0.232	0.261	5	0.644	0.769	0.162	0.131
East Yungui Plateau	4	62	8	0.712	0.845	0.158	0.175	7	0.271	0.277	0.022	0.035
South Yungui Plateau	5	70	11	0.620	0.804	0.229	0.368	5	0.215	0.232	0.075	0.082
Total	25	343	25	0.647	0.879	0.263	0.379	20	0.389	0.615	0.367	0.212

ITS, internal transcribed spacer; Pop No, the number of populations; Plant No, the number of plants; n, the number of chlorotypes/ribotypes.

**Table 2:** Analysis of molecular variance (AMOVA) of chloroplast haplotypes and ITS ribotypes in *D. officinale* populations.

Partitioning	Source of variation	df	cpDNA			ITS		
			SS	VC	PV (%)	SS	VC	PV (%)
**All regions**	Among population	24	132.158	0.35215 Va	34.02	49.05	0.12728 Va	29.76
	within population	318	217.227	0.68310 Vb	65.98	95.533	0.30042 Vb	70.24
	Total	342	349.385	1.03526		144.583	0.4277	
**Six regions**	Among regions	5	67.67	0.17983 Va	16.94	26.406	0.07257 Va	16.56
	Among populations	19	64.488	0.19842 Vb	18.7	22.644	0.06524 Vb	14.89
	within population	318	217.227	0.68310 Vc	64.36	95.533	0.30042 Vc	68.55
	Total	342	349.385	1.06136		144.583	0.43823	

**Table 3:** Summary of mismatch distribution parameters and neutrality tests for the two lineages (Clades III and IV) of *D. officinale* chloroplast haplotypes.

Model	Group	Parameter (τ)	SSD	P	H_Rag_	P	Fu’s F_S_	P	Tajima’s D	P
Spatial expansion	Clade III	—	—	—	—	—	0.745	N.A.	0.685	0.903
	Clade IV	1.510	0.044	0.193	0.262	0.327	0.789	N.A.	0.857	0.872
	All	2.173	0.063	0.292	0.242	0.349	0.860	0.803	0.709	0.567
Demographic expansion	Clade III	—	—	—	—	—	0.745	N.A.	0.685	0.902
	Clade IV	1.678	0.056	0.259	0.262	0.262	0.789	N.A.	0.857	0.877
	All	1.822	0.042	0.234	0.242	0.381	0.860	0.803	0.709	0.564
