# Supplementary material for: Iteration expansion and regional evolution: phylogeography of Dendrobium officinale and four related taxa in southern China
Source: Sci Rep. 2017 Mar 6;7:43525. doi: 10.1038/srep43525 (PMC5337965; doi:10.1038/srep43525)
Supplement: Supplementary Information [file srep43525-s1.pdf]

Iteration expansion and regional evolution: phylogeography of *Dendrobium officinale* and four related taxa in southern China

Beiwei Hou[1,2], Jing Luo[1], Yusi Zhang[1,3], Zhitao Niu[1], Qingyun Xue[1] and Xiaoyu Ding[1]*

[1]College of Life Sciences, Nanjing Normal University, Nanjing 210023, China

[2]Nanjing Institute for Comprehensive Utilization of Wild Plants, Nanjing 210042, China

[3]Jiangsu Industrial Technology Research Institute, Nanjing 210042, China

*Correspondence Xiao-Yu Ding, E-mail: dingxynj@263.net

Fig. S1 Distribution of the number of pairwise nucleotide differences for chloroplast (cp) DNA sequence data in *D. officinale* as a whole (A), and in its Clade III (B) and Clade IV (C)

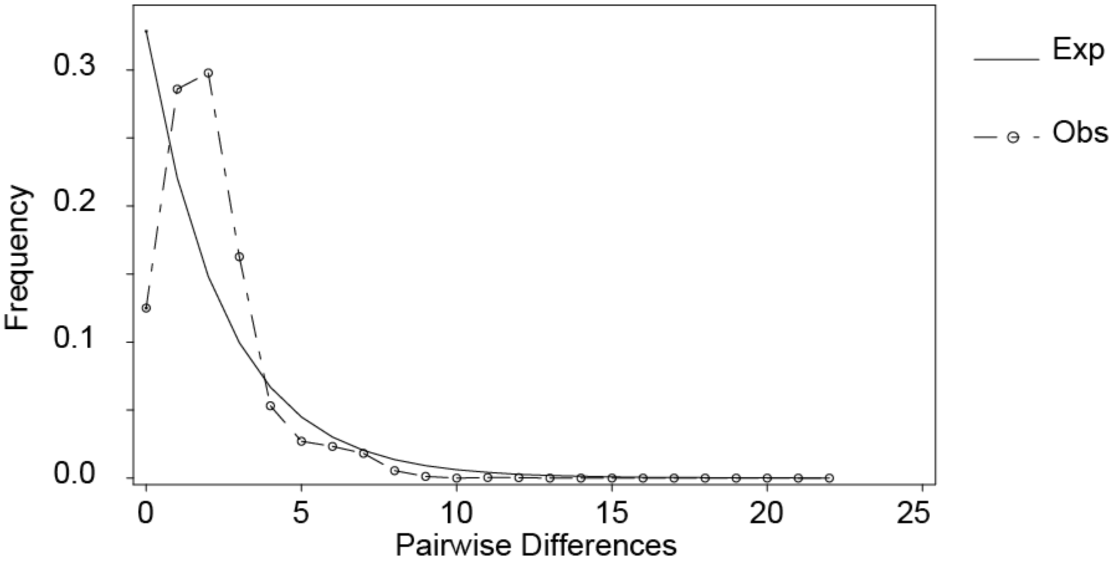

(A)

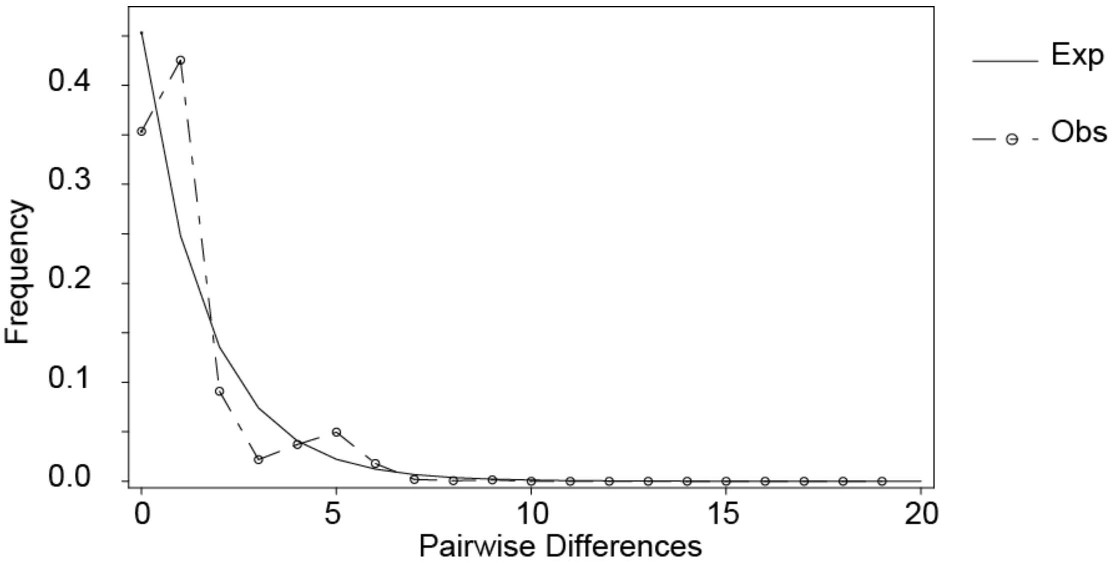

(B)

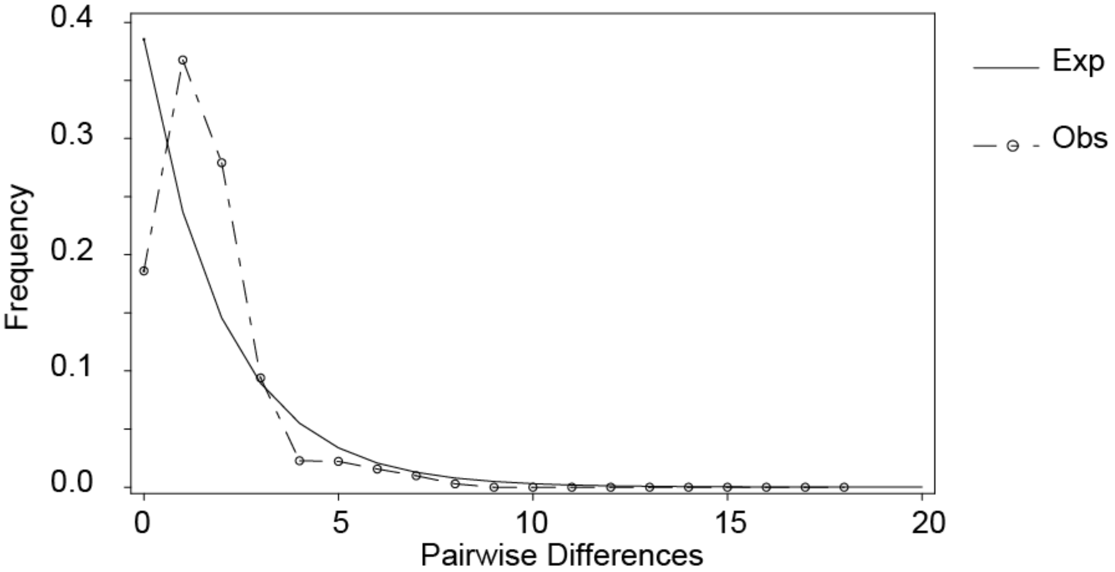

Frequency
0.4
0.3
0.2
0.1
0.0
0
5
10
15
20
Pairwise Differences
Exp
Obs

(C)

Table S1 Populations of *D. officinale* and four related taxa and their collection localities

POP	Population code	Locality	Population size	Latitude (°N)	Longitude (°E)
D. officinale					
**Yandang Mts.**					
pop01	ZJ-YD	Yandang, Zhejiang Province	16	28.36	121.06
pop02	ZJ-YJ	Yongjia, Zhejiang Province	15	28.17	120.71
pop03	ZJ-TZ	Taizhou, Zhejiang Province	12	28.72	120.97
pop04	ZJ-JH	Jinhua, Zhejiang Province	14	28.85	119.52
**Wuyishan Mts.**					
pop05	FJ-WY	Wuyi, Fujian province	14	27.87	117.78
pop06	FJ-SC	Shunchang, Fujian Province	14	26.67	117.77
pop07	FJ-LC	Liancheng, Fujian Province	14	25.71	116.81
pop08	JX-NF	Nanfeng, Jiangxi Province	12	27.21	116.38
pop09	JX-YT	Yingtan, Jiangxi Province	6	27.96	117.08
**Dabieshan Mts.**					
pop10	HB-YS	Yingshan, Hubei Province	10	30.71	115.76
pop11	AH-HS	Huoshan, Anhui Province	12	31.71	116.03
**Nanling Mts.**					
pop12	JX-JG	Jinggangshan, Jiangxi Province	10	26.73	114.21
pop13	GX-GL	Guilin, Guangxi Province	18	25.71	110.80
pop14	HN-CZ	Chenzhou, Hunan Province	14	25.72	113.22
pop15	GD-SG	Shaoguan, Guangdong Province	18	24.86	113.84
pop16	GD-PY	Pingyuan, Guangdong Province	12	24.63	115.74
**East Yungui Plateau**					
pop17	GZ-SD	Sandu, Guizhou Province	18	25.99	107.95
pop18	GZ-LB	Libo, Guizhou Province	16	25.32	107.92
pop19	GX-HC	Hechi, Guangxi Province	12	24.65	107.84
pop20	GX-TE	Tian'e, Guangxi Province	16	25.03	107.15
**South Yungui Plateau**					
Pop21	GX-XY	Xingyi, Guizhou Province	16	25.04	104.83
pop22	GX-XL	Xilin, Guangxi Province	14	24.54	105.05
Pop23	YN-GN	Guangnan, Yunnan Province	14	24.11	105.03
Pop24	YN-SP	Shiping, Yunnan Province	11	23.83	102.54
Pop25	YN-WS	Wenshan, Yunnan Province	15	23.32	104.12
D. tosaense					

**Nanling Mts.**					
DT-1	DT-LN	Longnan, Jiangxi Province	20	24.93	114.65
**Taiwan**					
DT-2	DT-TW	Taiwan	8	23.43	120.81
D. flexicaule					
**Funiushan Mts.**					
DF-1	DF-NZ	Nanzhao, Henan Province	16	33.67	112.39
**Daba Mts.**					
DF-2	DF-SN	Shennongjia, Hubei Province	20	31.83	110.71
**Wuling Mts.**					
DF-3	DF-ES	Enshi, Hubei Province	20	30.51	109.32
**East Hengduan Mts.**					
DF-4	DF-GL	Ganluo, Sichuan Province	8	28.87	102.82
D. scoriarum					
**South Yungui Plateau**					
DG-1	DG-XY	Xingyi, Guizhou Province	24	24.98	104.81
DG-2	DG-WS	Wenshan, Yunnan Province	20	23.45	104.62
D. shixingense					
**Nanling Mts.**					
DS	DS-SX	Shixing, Guangdong Province	20	24.84	114.02

Table S2 CpDNA haplotype sequence polymorphisms detected in *D. officinale* and related taxa

	H1	H2	H3	H4	H5	H6	H7	H8	H9	H10	H11	H12	H13	H14	H15	H16	H17	H18	H19	H20	H21	H22	H23	H24	H25	H26	H27	H28	H29	H30	H31	H32	H33	H34
Nucleotide position																																		
*acc*D-*psa*I (838 bp)																																		
23	C	.	.	.	.	.	.	.	.	.	.	.	.	.	.	.	.	.	.	.	.	.	.	.	.	.	A	A	A	.	.	.	.	.
138	A	.	.	.	.	.	.	.	.	.	.	.	.	.	.	.	.	.	.	.	.	.	.	.	.	.	.	.	.	C	C	C	.	.
163	T	.	.	.	.	.	.	.	.	.	.	.	G	.	.	.	.	.	.	.	.	.	.	.	.	.	.	.	.	.	.	.	.	.
173	-	-	-	-	-	-	-	-	-	-	-	-	-	-	-	-	-	-	-	-	-	-	-	-	-	-	1[a]	1[a]	1[a]	-	-	-	-	-
226	T	.	.	.	.	.	.	G	.	.	.	.	.	.	.	.	.	.	.	.	.	.	.	.	.	.	.	.	.	.	.	.	.	.
302	C	.	.	.	.	.	.	.	.	.	.	.	A	.	.	.	.	.	.	.	.	.	.	.	.	.	.	.	.	.	.	.	.	.
303	T	.	.	.	.	.	.	.	.	.	.	.	C	.	.	.	.	.	.	.	.	.	.	.	.	.	.	.	.	.	.	.	.	.
328	C	.	.	.	.	.	.	.	.	.	.	.	.	.	.	.	.	.	.	.	.	.	.	.	.	.	.	.	.	.	.	.	.	A
349	A	.	.	.	.	.	.	.	.	.	.	.	.	.	.	.	.	.	.	.	.	.	.	.	.	.	C	C	C	.	.	.	.	.
365	A	.	.	.	.	.	.	.	.	.	.	.	.	.	.	.	C	.	.	.	.	.	.	.	.	.	.	.	.	.	.	.	.	.
382	T	.	.	.	.	.	.	.	.	.	.	.	.	.	.	.	.	.	.	.	.	.	.	.	.	.	G	G	G	.	.	.	.	.
399	A	.	.	.	.	.	.	.	.	.	.	.	G	.	.	.	.	.	.	.	.	.	.	.	.	.	.	.	.	.	.	.	.	.
415	-	-	-	-	-	-	-	-	-	-	-	-	-	-	-	-	-	-	-	-	-	-	-	-	-	-	-	-	-	1[b]	1[b]	1[b]	-	-
446	A	.	.	.	.	.	.	.	.	.	.	.	.	G	.	.	.	.	.	.	.	.	.	.	.	.	.	.	.	.	.	.	.	.
471	T	.	.	.	.	.	.	.	.	.	.	.	.	.	.	.	.	.	.	.	.	.	.	.	.	.	.	.	.	G	G	G	.	.
506	T	.	.	.	.	C	.	.	C	.	.	.	.	.	.	.	.	.	.	.	.	.	.	.	.	.	.	.	.	.	.	.	.	.
577	2[a]	2[b]	2[b]	.	2[b]	.	2[b]	.	.	.	.	.	.	.	2[b]	.	2[b]	2[b]	2[b]	.	2[b]	2[b]	2[b]	.	.	.	2[b]	2[b]	2[b]	.	.	.	.	.
671	G	.	.	.	.	.	.	.	.	.	.	.	.	.	.	.	.	.	.	.	.	.	.	.	.	.	T	T	T	.	.	.	.	.

795 T	.	.	.	.	.	.	.	.	.	.	.	.	.	.	G	.	.	.	.	.	.	.	.	.	.	.	.	.	.	.	.	.	.
807 A	.	.	.	.	.	.	.	.	.	.	.	.	.	.	.	.	.	.	C	.	.	.	.	.	.	.	.	.	.	.	.	.	.
*trn*C-*pet*N (817 bp)																																	
38 C	.	.	.	.	.	.	.	.	.	.	.	.	.	.	.	.	.	.	.	.	T	.	.	.	.	.	.	.	.	.	.	T	.
62 C	.	.	.	.	.	.	.	.	.	.	.	.	.	.	.	.	.	.	.	.	T	.	.	.	.	.	.	.	.	.	.	.	.
95 C	.	G	.	.	.	.	.	.	.	.	.	.	.	.	.	.	.	.	.	.	.	T	.	.	.	.	.	.	.	.	.	.	.
170 C	.	A	A	A	.	A	A	.	.	A	.	.	.	.	A	.	A	A	.	A	A	A	A	A	.	.	.	.	.	.	.	.	.
237 G	.	.	.	.	.	.	.	.	.	.	.	.	.	.	.	.	.	.	.	.	.	.	.	.	.	.	.	.	.	.	.	T	T
297 G	.	.	.	.	.	.	.	.	.	.	.	.	.	A	.	.	.	.	.	.	.	.	.	.	.	.	.	.	.	.	.	.	.
315 T	.	.	.	.	.	.	.	.	.	.	.	.	.	.	.	.	.	.	.	.	.	.	.	.	G	.	.	.	.	.	.	.	.
353 C	.	.	.	.	.	.	.	.	.	.	.	.	.	.	.	.	.	.	.	.	.	.	.	.	T	.	.	.	.	.	.	.	.
357 G	.	.	.	.	.	.	.	.	.	.	.	.	.	.	.	.	.	.	.	.	.	.	.	.	.	.	.	.	.	.	.	A	A
422 A	.	.	.	.	.	.	.	.	.	.	.	.	.	.	.	.	.	.	.	C	.	.	.	.	.	.	.	.	.	.	.	.	.
433 A	.	.	.	.	.	.	.	.	.	.	.	.	.	.	.	.	.	.	.	C	.	.	.	.	.	.	.	.	.	.	.	.	.
460 T	.	.	.	.	.	.	.	.	.	.	.	.	.	.	.	.	.	.	.	.	.	.	.	.	.	.	.	G	.	.	.	.	.
487 A	.	.	.	.	.	.	.	.	.	.	.	.	.	.	.	.	.	.	.	.	.	.	.	.	.	.	.	G	.	.	.	.	.
488 G	.	.	.	.	.	.	.	.	A	.	.	.	.	.	.	.	.	.	.	.	.	.	.	.	.	.	.	A	.	.	.	.	.
518 A	.	.	.	.	.	.	.	.	.	.	.	.	.	.	.	.	.	.	.	.	.	.	.	.	C	.	.	.	.	.	.	.	.
550 C	.	.	.	.	.	.	.	G	.	.	.	.	.	.	.	.	.	.	.	.	.	.	.	.	.	.	.	.	.	.	.	.	.
553 A	.	.	.	.	.	.	.	G	.	.	.	.	.	.	.	.	.	.	.	C	.	.	.	.	.	.	.	.	.	.	.	.	.
556 A	.	.	.	.	.	.	.	T	.	.	.	.	.	.	.	.	.	.	.	.	.	.	.	.	.	.	.	.	.	.	.	.	.
561 C	.	.	.	.	.	.	.	.	.	.	.	.	.	.	.	.	.	.	.	.	.	.	T	.	.	.	.	.	.	.	.	.	.
563 C	.	.	.	.	.	.	.	.	T	.	.	.	.	.	.	.	.	.	.	.	.	.	.	.	.	.	.	.	.	.	.	.	.
589 G	.	.	.	.	.	.	.	.	.	.	.	.	.	A	.	.	.	.	.	.	.	.	.	.	.	.	.	.	.	.	.	.	.
592 T	.	.	.	.	.	.	.	A	.	.	.	.	.	.	.	.	.	.	.	.	.	.	.	.	.	.	.	.	.	.	.	.	.
616 C	.	.	.	.	.	.	A	.	.	.	.	.	.	.	.	.	.	.	.	.	.	.	.	A	.	.	.	.	.	.	.	.	.
627 T	.	.	.	.	.	A	.	.	.	.	.	.	.	.	.	.	A	.	.	.	.	.	.	.	.	.	.	.	.	.	.	.	.

6 6 5	G	.	.	.	.	.	.	.	.	.	.	.	.	.	.	.	.	.	.	.	.	A	.	.	.	.	.	.	.	.	A	A	.	.
6 7 8	T	.	.	.	.	.	.	.	.	.	.	G	.	.	.	.	.	.	.	.	.	.	.	.	.	.	.	.	.	.	.	.	.	.
6 8 1	G	.	.	.	.	.	.	.	.	.	.	.	.	.	.	.	.	.	.	.	.	A	.	.	.	.	.	.	.	.	A	A	.	.
7 4 2	G	.	.	.	.	.	.	.	.	.	.	.	.	.	.	.	.	.	.	.	.	A	.	.	.	.	.	.	.	.	.	A	.	.
*rps*15-*ycf*1 (542 bp)																																		
1 5 5	T	.	.	.	.	.	.	.	.	.	.	.	.	.	.	.	.	.	.	.	.	.	.	.	.	.	.	.	.	G	G	G	.	.
1 7 5	A	.	.	.	.	.	.	.	.	.	.	.	.	.	.	.	.	.	.	.	.	.	.	.	.	.	.	.	.	.	.	.	.	C
2 6 0	-	-	-	-	-	-	-	-	-	-	-	-	-	-	-	-	-	-	-	-	-	-	-	-	-	-	-	-	-	1^c	1^c	1^c	-	-
3 0 0	A	.	.	.	.	.	.	.	.	.	.	.	.	.	.	.	.	.	.	.	.	.	.	.	.	.	G	G	G	G	G	G	.	.
3 2 5	T	.	G	.	.	.	.	.	.	.	G	.	.	.	.	G	.	G	G	.	.	.	G	.	.	.	.	.	.	.	.	.	.	.
4 4 9	C	.	.	.	.	.	.	.	.	.	.	.	.	.	.	.	.	.	.	.	.	.	.	.	.	.	A	.	.	.	.	.	.	.
5 0 6	G	.	.	.	.	.	.	.	.	.	.	.	.	.	.	.	.	.	.	.	.	.	.	.	.	.	.	.	.	T	T	T	.	.
5 2 2	C	.	.	.	.	.	.	.	.	.	.	.	.	.	.	.	.	.	.	.	.	.	.	.	.	.	G	G	G	G	G	G	.	.

All sequences are compared to that of the reference haplotype H1. Note that poly-A or poly-T stretches were excluded from the analysis. A dash (-) denotes a single nucleotide indel. Numbers ‘0/1’ in the sequences indicate absence/presence of length polymorphisms: 1^a, CTAATGAAA; 1^b, AAAGAAAAT; 1^c, TATATTT. The number ‘2’ stands for presence of an inversion: 2^a, CCTTTTTT; 2^b, AAAAAGG.

Table S3 Summary of cpDNA haplotypes in populations of *D. officinale* and related taxa

Species	Populations	Clade I	Clade II	Clade III	Clade IV	Nc
D. flexicaule	DF1-2	H27, H28, H29				**3**
D. scoriarum	DG1-2		H31, H32, H33			**3**
D. shixingense	DS			H33, H34		**2**
D. tosaense	DT1-2			**H1**, H26		**2**
D. officinale						
Yandang Mts.	pop1-4			**H1**, **H2**, H6	**H4**, **H5**, H3, H7	**7**
	pop1			H1, H2	H3	3
	Pop2			H1, H2, H6	H4, H5, H7	6
	pop3			H1, H2, H6		3
	Pop4			H1, H2	H4, H5	4
Wuyishan Mts.	pop5-9			**H1**, **H2**, H9, H10	**H4**, **H5**, H8, **H11**	**8**
	pop5			H1, H2	H5	3
	pop6			H1	H4, H5, H8	4
	pop7			H1, H9, H10	H5	4

	pop8	H2,	H4, H5,	3
	pop9		H5, H11	2
Dabieshan Mts.	pop10-11	**H1**, H12, H13, H14		**4**
	pop10	H1, H12		2
	pop11	H1, H13, H14		3
Nanling Mts.	pop12-16	**H1**, **H2**, H15, H17, H20	**H4**, **H5**, H8, **H11**, H16, H18, **H19**	**12**
	pop12	H1, H15	H5	3
	pop13	H2, H17	H8, H16, H18	5
	pop14		H5, H11, H19	3
	pop15	H1, H2, H20	H4, H5,	5
	pop16	H2		1
East Yungui Plateau	pop17-20	**H1**, **H2**, H20	**H4**, **H5**, **H11**, H21, H22	**8**
	pop17	H2	H4, H5, H11	4
	pop18	H2	H5, H21	3
	pop19		H5, H11	2
	pop20	H1, H20	H4, H11, H22	5
South Yungui Plateau	pop21-25	**H2**	**H4**, **H5**, H3, H7, **H11**, H16, **H19**, H23, H24, H25	**11**

pop21		H11, H19	2
pop22	H2	H4, H11, H16, H19	5
pop23		H3, H19, H23	3
pop24		H11, H19	2
pop25		H4, H5, H7, H16, H24, H25	6

Nc, the number of haplotypes

Table S4 Summary of ITS ribotypes in populations of *D. officinale* and related taxa

Species	Populations	Clade A	Clade B	Nc
D. flexicaule	DF1-2	R21, R22		**2**
D. scoriarum	DG1-2	**R2**, R23, R24, R25		**4**
D. shixingense	DS	R24		**1**
D. tosaense	DT1-2		**R1**	**1**
D. officinale				
Yandang Mts.	pop1-4	**R2**, R3	**R1**	**3**
	pop1	R2	R1	2
	Pop2	R2, R3	R1	3
	pop3	R2	R1	2
	Pop4	R2, R3	R1	3
Wuyishan Mts.	pop5-9	**R2**, R9	**R1**, R4, R5, R6, R7, R8	**8**
	pop5	R2	R1, R4, R5	4
	pop6	R2	R6	2
	pop7	R2	R1, R7	3
	pop8	R2, R9	R1, R5, R8	5
	pop9	R2, R9	R1	3
Dabieshan Mts.	pop10-11	**R2**, R10, R11, R12	**R1**	**5**
	pop10	R10, R11	R1	3
	pop11	R2, R12		2
Nanling Mts.	pop12-16	**R2**, R13	**R1**, R5, R6	**5**
	pop12	R2, R13	R5	3
	pop13	R2	R1 , R6	3
	pop14	R2		1
	pop15	R2	R1, R5	3
	pop16		R1	1

East Yungui Plateau	pop17-20	**R2**, R9, R15, R16, R17	**R1**, R14	**7**
	pop17	R2, R9	R1, R14	4
	pop18	R2, R15	R1	3
	pop19	R2		1
	pop20	R16, R17		2
South Yungui Plateau	pop21-25	**R2**, R9, R19, R20	R18	**5**
	pop21	R2		1
	pop22	R2, R9	R18	3
	pop23	R2, R19		2
	pop24	R2, R20		2
	pop25	R2		1

Nc, the number of ribotypes

Table S5 Primers used to amplify target genes

Target gene	Primer sequence (5’-3’)
nrDNA ITS	F: CGTAACAAGGTTTCCGTAGGTGAAC
	R: TTATTGATATGCTTAAACTCAGCGGG
*acc*D-*psa*I	F: GGAAGTTTGAGCTTTATGCAAATGG
	R: AGAAGCCATTGCAATTGCCGGAAA
*trn*C-*pet*N	F: GACTGCAAATCCTTTATCC
	R: CAGGGGACTGCAAATCCTT
*rps*15-*ycf*1	F: TGTGAAGTAAGTCTCCGTATCT
	R: GCTTGTATGAATCGCTATTGGT

Table S6 Accession numbers of the sequences of four loci submitted to GenBank

Species	Haplotype	*acc*D-*psa*I	*trn*C-*pet*N	*rps*15-*ycf*1	Ribotype	ITS
D. officinale	H1	KT779634	KT779672	KT779710	R1	KT779748
	H2	KT779635	KT779673	KT779711	R2	KT779749
	H3	KT779636	KT779674	KT779712	R3	KT779750
	H4	KT779637	KT779675	KT779713	R4	KT779751
	H5	KT779638	KT779676	KT779714	R5	KT779752
	H6	KT779639	KT779677	KT779715	R6	KT779753
	H7	KT779640	KT779678	KT779716	R7	KT779754
	H8	KT779641	KT779679	KT779717	R8	KT779755
	H9	KT779642	KT779680	KT779718	R9	KT779756
	H10	KT779643	KT779681	KT779719	R10	KT779757
	H11	KT779644	KT779682	KT779720	R11	KT779758
	H12	KT779645	KT779683	KT779721	R12	KT779759
	H13	KT779646	KT779684	KT779722	R13	KT779760
	H14	KT779647	KT779685	KT779723	R14	KT779761
	H15	KT779648	KT779686	KT779724	R15	KT779762
	H16	KT779649	KT779687	KT779725	R16	KT779763
	H17	KT779650	KT779688	KT779726	R17	KT779764
	H18	KT779651	KT779689	KT779727	R18	KT779765
	H19	KT779652	KT779690	KT779728	R19	KT779766
	H20	KT779653	KT779691	KT779729	R20	KT779767

	H21	KT779654	KT779692	KT779730		
	H22	KT779655	KT779693	KT779731		
	H23	KT779656	KT779694	KT779732		
	H24	KT779657	KT779695	KT779733		
	H25	KT779658	KT779696	KT779734		
D. tosaense	H1	KT779659	KT779697	KT779735	R1	KT779768
	H26	KT779660	KT779698	KT779736		
D. flexicaule	H27	KT779661	KT779699	KT779737	R21	KT779769
	H28	KT779662	KT779700	KT779738	R22	KT779770
	H29	KT779663	KT779701	KT779739		
D. scoriarum	H30	KT779664	KT779702	KT779740	R2	KT779771
	H31	KT779665	KT779703	KT779741	R23	KT779772
	H32	KT779666	KT779704	KT779742	R24	KT779773
					R25	KT779774
D. shixingense	H33	KT779667	KT779705	KT779743	R24	KT779775
	H34	KT779668	KT779706	KT779744		
D. linawianum	-	KT779669	KT779707	KT779745	-	KT779776
D. moniliforme	-	KT779670	KT779708	KT779746	-	KT779777
	-	KT779671	KT779709	KT779747	-	KT779778
